# A Low Complexity Efficient Deep Learning Model for Automated Retinal Disease Diagnosis

**DOI:** 10.1007/s41666-024-00182-5

**Published:** 2025-01-03

**Authors:** Sadia Sultana Chowa, Md. Rahad Islam Bhuiyan, Israt Jahan Payel, Asif Karim, Inam Ullah Khan, Sidratul Montaha, Md. Zahid Hasan, Mirjam Jonkman, Sami Azam

**Affiliations:** 1 Faculty of Science and Technology, Charles Darwin University, Casuarina, NT 0909 Australia; 2 Health Informatics Research Laboratory (HIRL), Department of Computer Science and Engineering, Daffodil International University, Dhaka-1341, Bangladesh; 3 Department of Computer Science, University of Calgary, Calgary, AB T2N 1N4 Canada

**Keywords:** Optical coherence tomography (OCT), Retinal disease, Compact convolutional transformer (CCT), Transformer model, Generative adversarial network (GAN), Ablation studies

## Abstract

The identification and early treatment of retinal disease can help to prevent loss of vision. Early diagnosis allows a greater range of treatment options and results in better outcomes. Optical coherence tomography (OCT) is a technology used by ophthalmologists to detect and diagnose certain eye conditions. In this paper, human retinal OCT images are classified into four classes using deep learning. Several image preprocessing techniques are employed to enhance the image quality. An augmentation technique, called generative adversarial network (GAN), is utilized in the Drusen and DME classes to address data imbalance issues, resulting in a total of 130,649 images. A lightweight optimized compact convolutional transformers (OCCT) model is developed by conducting an ablation study on the initial CCT model for categorizing retinal conditions. The proposed OCCT model is compared with two transformer-based models: vision Transformer (ViT) and Swin Transformer. The models are trained and evaluated with 32 × 32 sized images of the GAN-generated enhanced dataset. Additionally, eight transfer learning models are presented with the same input images to compare their performance with the OCCT model. The proposed model’s stability is assessed by decreasing the number of training images and evaluating the performance. The OCCT model’s accuracy is 97.09%, and it outperforms the two transformer models. The result further indicates that the OCCT model sustains its performance, even if the number of images is reduced.

## Introduction

The retina, an intricate eye component, converts light into signals that the brain can comprehend. The photosensitive layer, which lines the eyeball’s inner surface, receives the light that is focused by the lens and converts it into neural impulses [1]. The retina is considered the most crucial component of the eye [2]. However, many people are affected by retinal disorders, particularly the elderly and people with underlying systemic illnesses like diabetes [3]. There are some common eye diseases which affect the retina such as diabetic macular edema (DME), choroidal neovascularization (CNV), and Drusen. A non-invasive imaging method, known as optical coherence tomography (OCT), can assist in diagnosing such retinal problems. Identifying retinal abnormalities requires skill, is time-consuming, and may result in misdiagnosis [4]. Artificial intelligence (AI) is capable of handling disease categorization procedures that are time-consuming and laborious for specialists [5]. This research aims to develop an automated decision support system for retinal disease identification which is efficient and reliable.

Medical images may differ in terms of appearance, size, shape, and orientation. This can make it challenging for deep learning models to classify or generalize unseen images. A dataset containing noisy images and an imbalanced distribution of classes may cause biases in the deep neural network’s training process [6]. So, for better model performance, image processing and augmentation are essential for such type of dataset. High-dimensional data is frequently included in medical imaging, requiring a lot of computational resources and parameters to process. Reducing the number of pixels in the images may shorten the training period. However, it is a matter of concern that training with low dimensional images may also yield poor performance. To overcome the issue, a CCT model can be useful as it combines and utilizes the benefits of convolutional neural networks (CNN) and transformers to analyze medical images efficiently and provide improved performance, making it a promising choice for medical image datasets.

Our research aims to classify retinal disease using OCT images with a deep transformer learning model CCT, utilizing data augmentation. The dataset is enhanced through a generative adversarial network (GAN) prior to the model generation. Additionally, a variety of image preprocessing techniques, to improve the performance, are assessed. The CCT model is subjected to an ablation study for seven variables which results in the OCCT model, the model with the highest accuracy. The ViT and Swin Transformer models are trained with the same optimizer, activation function, loss function, pooling layer, and learning rate as the fine-tuned OCCT model, and their performance is compared. Additionally, the OCCT, ViT, and Swin Transformer models were assessed for their resilience by using a reduced set of images for both real and enhanced OCT datasets. Furthermore, we have presented that a transformer-based architecture can outperform CNN models like DenseNet121, DenseNet201, ResNet50, MobileNetV2, ResNet101V2, VGG16, VGG19, and EfficientNetB1 with 32 × 32 image size. *K*-fold cross-validation and confusion matrix evaluation are conducted to assess the performance consistency of the model. Moreover, to demonstrate the efficacy of GAN and proposed model, performance comparisons with prior studies are carried out for both.

The major contribution of this study is summarized as follows:

- An optimized transformer model, OCCT, is introduced that is configured based on a comprehensive ablation study consisting of seven experiments.
- An automatic augmentation method is proposed utilizing GAN to address the issue of class imbalance within the dataset.
- A performance comparison is done with two transformer models and eight transfer learning models with the same configuration as the proposed model and the OCCT model consistently outperform all other models.
- An experiment is carried out between two transformer model, ViT and Swin, and the proposed model by gradually decreasing the number of images to assess the model’s reliability with limited image data, where the proposed model shows an exceptional performance even with a reduced number of images.

## Literature Review

The advancement of retinal disease diagnosis is pursued by implementing different Machine learning and deep learning models. Rajagopalan et al. [2] developed a CNN classification scheme for three different retinal diseases. It was trained using 12,000 images. The network received input images with spatial dimensions of 224 × 224 × 1, using denoised images, and an eleven-layer CNN architecture was used to categorize retinal abnormalities. The accuracy was 97.01%. Alqudah et al. [1] worked with a novel automated CNN architecture for a spectral-domain optical coherence tomography-based multiclass classification system (SD-OCT). 108,312 retinal OCT images in five different classes were included in their dataset. They introduced a newly designed algorithm to train the deep network architecture’s neuron layers (greedy layer-wise training). By implementing the Adam optimization technique, the neural network attained an accuracy of 98.1%, a sensitivity of 97.12%, and a specificity of 99.28%. Tayal et al. [5] proposed a deep-learning-based diagnostic tool for four-class classification DME, Drusen, CNV, and normal OCT images. Retinal OCT images underwent noise elimination, contrast enhancement, identification of edges based on contours, and extraction of retinal layers. The study employed three distinct CNN-based model designs, namely a five-layer CNN model, a seven-layer CNN model, and a nine-layer CNN model. The seven-layer CNN model had a 96.5% accuracy. Rajagopalan et al. [7] developed a deep CNN framework for diagnosis and categorization into normal, DMD, and DME. To decrease any intrinsic speckle noise in the input OCT images, a Kuan filter was applied. Hyperparameter optimization was utilized to optimize the CNN network. The proposed model had a 95.7% accuracy. Singh et al. [3] aimed to establish an explainable deep learning approach for retinal OCT diagnosis. The study tested and assessed explainable deep learning algorithms for detecting three retinal diseases: CNV, DME, and Drusen. Before the evaluation phase, the model was trained on 84,000 images and subsequently tested on a set of 1000 images, with 250 images per class. Chen et al. [8] evaluated different approaches to diagnose age-related macular degeneration (AMD) and DME. The VGG19, Resnet101, and Resnet50 models performed remarkably well in categorizing OCT images into AMD and DME when appropriate algorithm hyperparameters were used. Ai et al. [4] developed a hybrid attention technique to categorize and distinguish retinopathy (Drusen, CNV, and DME) images. Using two public OCT datasets and a hybrid attention mechanism, which combines a parallel spatial attention mechanism with a channel attention mechanism, they obtained classification accuracies of 96.5% and 99.76%. Kayadibi et al. [9] introduced a hybrid methodology that combines dual preprocessing and a fully dense fusion neural network (FD-CNN) to detect retinal diseases. In the dual preprocessing, noise in OCT images is diminished through the application of a hybrid speckle reduction filter. Following the training of the FD-CNN architecture, features are extracted. In order to reclassify these features, deep support vector machine (D-SVM) and deep K-nearest neighbor (D-KNN) classifiers are implemented. In both datasets, D-SVM performed best, with 99.60% accuracy, 99.60% sensitivity, 99.87% specificity, 99.60% precision, and 99.60% F1 score in the UCSD dataset. In the Duke dataset, it achieved 97.50% accuracy, 97.64% sensitivity, 98.91% specificity, 96.61% precision, and 97.03% F1 score. Hassan et al. [10] presented an EOCT model that improves the classification of retinal OCT images by employing a random forest and modified ResNet (50) algorithms. The study incorporated the Adam optimizer during the training phase with the aim of improving the performance of ResNet (50) relative to conventional pre-trained models. The results of the experiments demonstrate that the EOCT model that was proposed achieved significant enhancements in the following performance metrics: accuracy (97.47%), sensitivity (98.36%), specificity (96.15%), precision (97.40%), negative predictive value (97.56%), false discovery rate (3.85%), and false negative rate (2.6%). Mathews et al. [11] presented an automated diagnostic system that utilizes deep learning to identify diabetic macular edema (DME) and Drusen macular degeneration (DMD). The Mendeley OCT and Duke datasets are used to test the suggested model, which is made up of residual blocks and channel attention modules. When applied to the Mendeley test dataset, the results show an impressive classification accuracy of 99.5%. When applied to the Duke dataset, the results show an accuracy of 94.9%. A thorough study of pre-trained models shows that the suggested model outperforms existing methods, even though it has fewer trainable parameters. Karthik et al. [12] proposed a standard ResNet architectures’ residual connection which was replaced with featuring an activation function that preserves negative weights and reinforces smaller gradients. The proposed design enhanced the classification accuracy in lab conditions, especially when baseline accuracy is over 98% (< 1% gain) or below (1.6% gain). Two OCT datasets with four and eight disease classifications were used. With OCT-C4 data, ablation studies show an average accuracy loss of 0.875%, and with OCT-C8 data, an accuracy loss of 1.39%. Jin et al. [13] introduced a two-stage deep learning (DL) system designed for accurate automatic grading of ERM. With its training on human-segmented core features, iERM improves classification performance and makes the data more comprehensible. iERM outperforms conventional DL models in grading performance by a significant margin (1–5.9%). The model attains exceptionally high accuracy ratings of 82.9%, 87.0%, and 79.4%, respectively, on the internal test dataset and two external test datasets. While the literature offers a diverse range of methodologies for retinal disease classification, this study presents a significant advancement with a lightweight OCCT model, demonstrating enhanced accuracy and stability with reduced training data.

## Method

### Dataset Description

The dataset includes a total of 108,312 images, of which 37,206 are CNV images, 11,349 DME images, 8617 Drusen images, and 51,140 normal images. The dataset is created by Zhang Lab at the University of California at San Diego (UCSD) and is available for free at Mendeley [14]. Each class refers to a different condition of the retina [15] (see Table 1).

**Table 1:** Different conditions of the retina for each class

Class	Description
CNV	Blood vessels in the sub-retinal spaces
DME	The described condition is a consequence of diabetes, in which fluid builds up in the macula, the core area of the retina responsible for clear vision. It can cause blindness
Drusen	Drusen are yellow deposits under the retina. Drusen are made up of lipids and proteins and can cause central vision loss
Normal	Images of a normal healthy retina

**Fig. 1:**
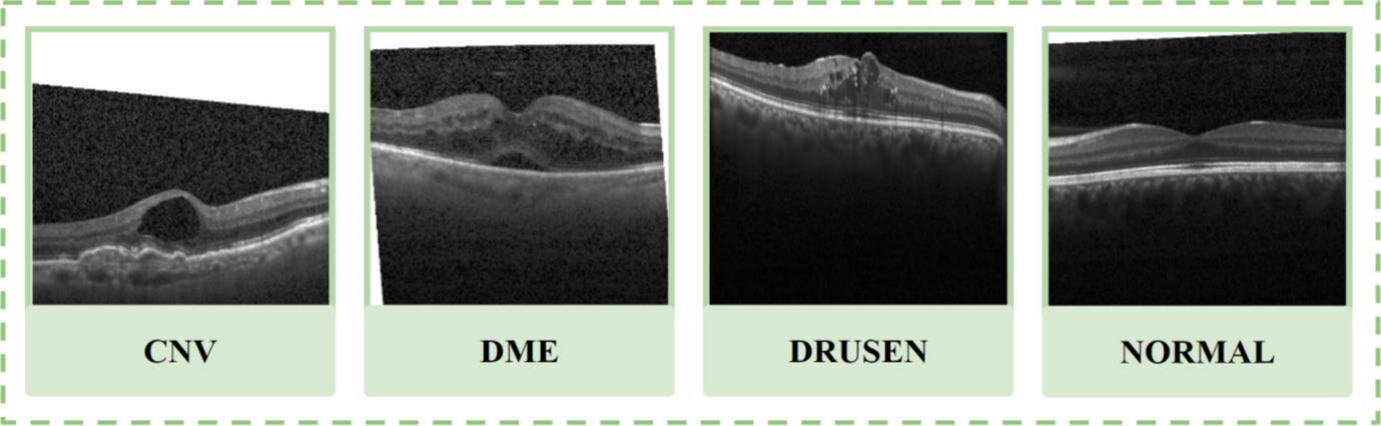
Different types of conditions of the retina

Figure 1 shows OCT images in each of the four classes.

**Fig. 2:**
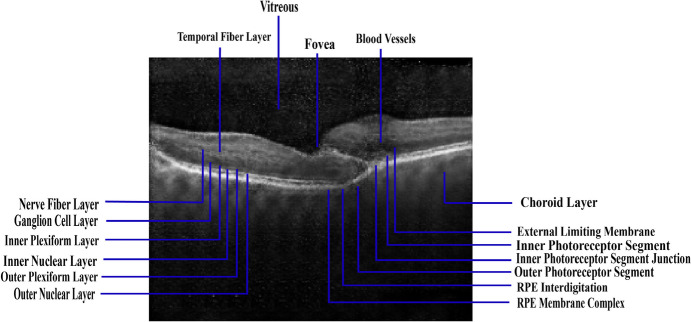
Labeled structure of an OCT image of a healthy normal retina

There are multiple distinct layers visible in an OCT retinal image. A labeled structure of an OCT image of a healthy normal retina is depicted in Fig. 2. The retina comprises various layers, including the outer nuclear layer (ONL), retinal nerve fiber layer (RNFC), ganglion cell layer and inner plexiform layer (GCL + IPL), inner nuclear layer (INL), inner segmentation and outer segmentation of photo receptor layer with junction, outer plexiform layer (OPL), retinal pigment epithelium layer (RPE), and RPE complex [16]. The retinal nerve fiber layer (RNFL) transfers visual input from the eye to the brain. The RNFL can provide crucial diagnostic data about eye health and the progression of eye disorders. Measurements of the RNFL thickness can be used to identify early stages of eye conditions and to monitor the development over time. The ganglion cell layer (GCL) accommodates the cell bodies of retinal ganglion cells that interpret visual data and transmit it to the brain through the optic nerve. The IPL contains the dendrites and axons of bipolar and amacrine cells, which help to refine and modulate visual signals before they are transmitted to the ganglion cells. The thickness and integrity of these layers can provide important diagnostic information about the health of the eye and the status of various eye diseases. The ganglion cells receive visual signals from the photoreceptor cells which are modified and modulated by the INL. Because INL damage can develop early in the course of a disease, it is an important target for early detection and treatment. The OPL is essential to get visual information from the photoreceptor cells to the bipolar cells. Because damage to the OPL can occur at an early stage of the disease process, it is also an important target for early identification and intervention. The ONL is essential for the initial processing of visual information. Loss of vision can be the consequence of damaged or dying photoreceptor cells in the ONL. The light that enters the eye is converted into electrical signals that other retinal cells can understand by photoreceptor cells in the ISL and OSL. As the photoreceptor layer can get damaged, leading to visual loss that may begin early in the disease process, it is also a key target for early identification and management of retinal illnesses. The primary role of the RPE is to absorb extraneous light that enters the eye and stop it from bouncing around and harming other retinal cells. The RPE is a primary target for retinal diseases such as age-related macular degeneration. A variety of visual problems can be caused by injury or abnormalities in any of the layers of the retina. The indications and manifestations may vary, contingent upon the layer of the retina that has been damaged.

### Methodology

**Fig. 3:**
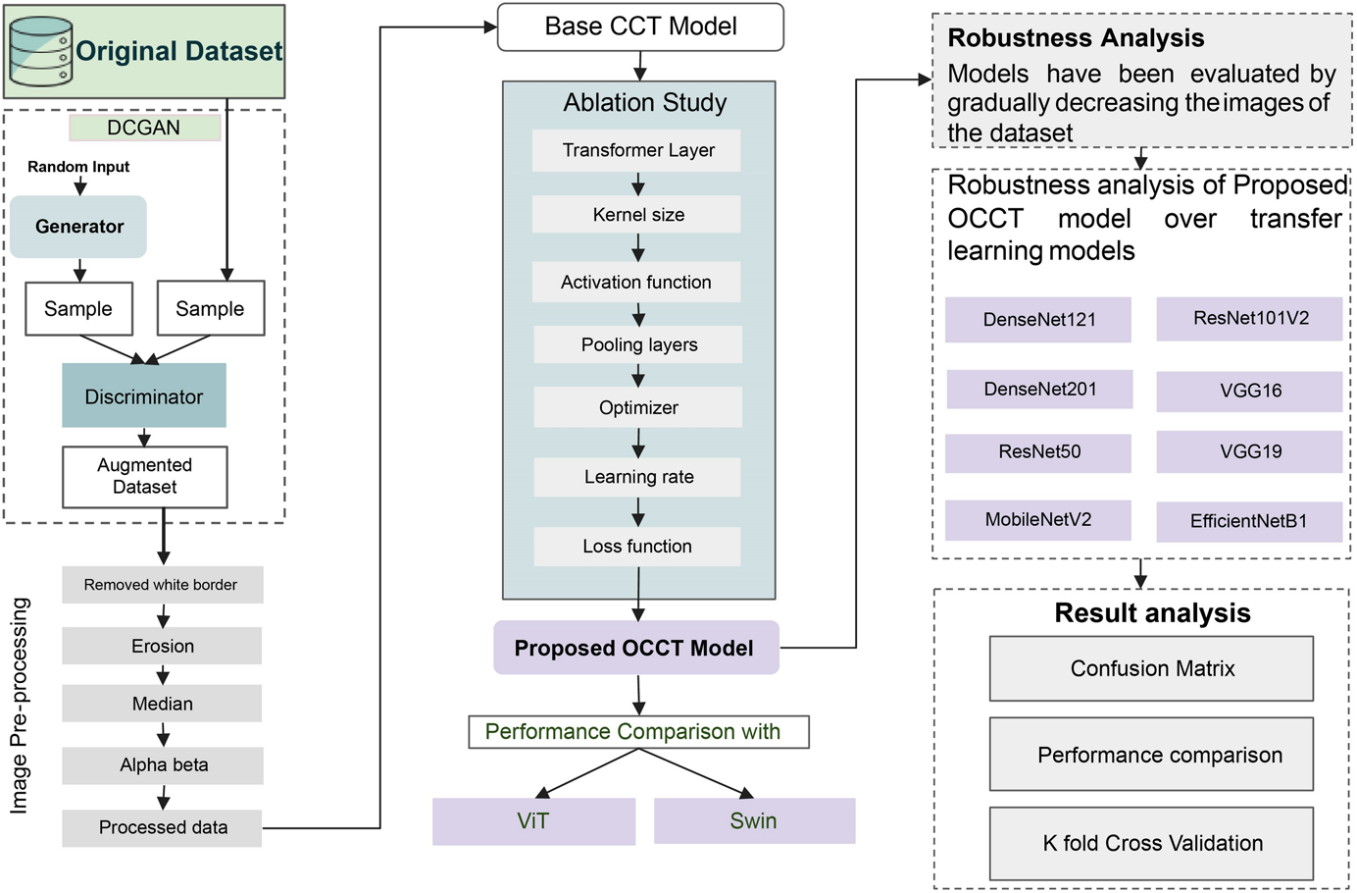
The classification process for retinal OCT images

An overview of the method utilized in this research is depicted in Fig. 3.

The dataset consists a total of 108,312 images in four classes. Since the dataset is imbalanced regarding the number of images in each class, a data augmentation technique named GAN is applied. Then, a preprocessed dataset is generated by applying several image preprocessing techniques on the augmented dataset. This includes removing artifacts and improving the quality of the images, using white border removal, erosion, median, and alpha beta correction. The proposed OCCT model is developed by performing with seven ablation studies where the model’s layer design and hyperparameters are altered. In order to reduce the numbers of parameters and the time complexity, and improve the performance, ablation studies based on the transformer layer, kernel layer, activation function, pooling layers, learning rate, optimizer, and loss function are carried out. The OCCT model’s performance is evaluated through a comparison with the ViT and Swin transformers. In addition, the robustness of the model is evaluated by gradually decreasing the number of images. Additionally, the proposed model’s performance is compared to transfer learning models like DenseNet201, ResNet50, MobileNetV2, ResNetV2, VGG16, VGG19, and EfficientNetB1. Lastly, several statistical analyses, confusion matrix, and *K*-fold cross-validation are evaluated to assess the performance consistency of the proposed model.

### Data Augmentation Using Deep Convolutional GAN (DCGAN)

In computer vision, especially in the medical domain, there is often a scarcity of images. This makes it difficult to accurately predict the classes. Augmenting data aids in amplifying the quantity of images and facilitates the training of the model by presenting samples that the model has not encountered previously. A popular augmentation approach, called GAN, produces new data that share the same traits as the training data. It is basically a deep CNN and can also be called DCGAN. It can utilize both supervised and unsupervised learning [17]. GANs have therefore become a focus point of research in computer vision.

### DCGAN

GAN is a machine learning technique that automatically discovers and learns the patterns of the input data and generates samples, maintaining similarity to the original dataset. Using a GAN, the probability density function need not be explicitly modeled to generate data [18]. The architecture of a GAN typically involves two components: a generator and a discriminator [19, 20]. The generator leverages a random input vector to produce a counterfeit sample that emulates the characteristics of the original data. The discriminator predicts whether the sample is real or not, after being trained with both real and fake sample data [21]. If the discriminator detects any false sample data, it returns those false data to the generator. The generator then creates improved false data which is sent back to the discriminator for recognition. The generator and detector must constantly develop themselves to enhance their respective generational and discriminative abilities. The loss function’s minimization enhances the generator’s performance, while the loss function’s maximization enhances the discrimination’s performance.

The primary distinction between DCGAN and previously developed GANs is that the DCGAN utilizes a CNN. To train the generator and discriminator networks, Eq. 1 is used [22].

1 $$V(R, C)= {E}_{x\sim {P}_{data}(x)}\left[logR\left(x\right)\right]+ {E}_{z\sim nz}\left[log\left(1-R\left(C\left(z\right)\right)\right)\right]$$

**Fig. 4:**
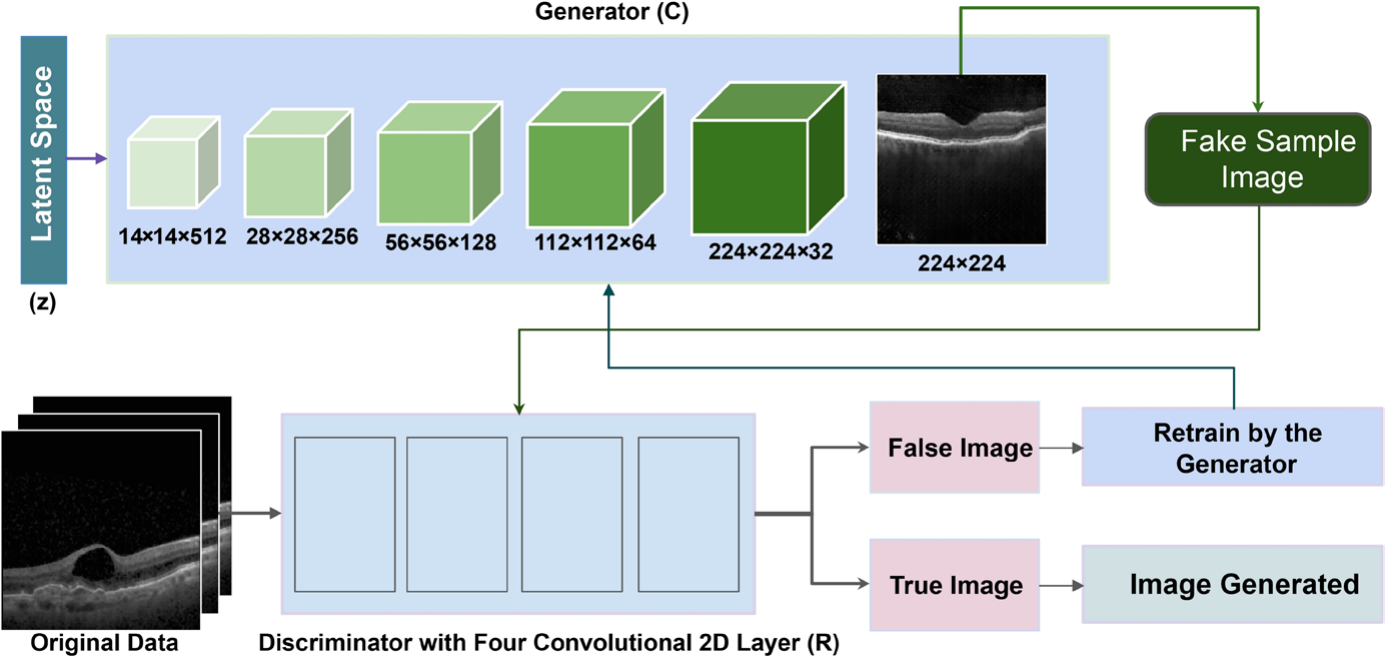
DCGAN architecture

Here, *R* and *C* represent the discriminator and generator, *x* denotes the original data, and *z* is the latent space, which is a hidden representation of data points. *R(x)* stands for the probability of *x*, which is sampled from real data, over the generated data, and *C(z)* denotes the generated data. ${E}_{x\sim {P}_{data}(x)}$ represents the expected values for both real and fake cases, and ${E}_{z\sim nz}$ represents the overall anticipated values where *nz* denotes the random noise variable. To maximize the accuracy of the discriminator (*R*), it is trained to accurately discriminate the real data (*x*) from the generated data (G(z)), minimizing $log\left(1-R\left(C\left(z\right)\right)\right)$ [22]. Figure 4 shows the DCGAN architecture.

As the generator’s initial input, we have used a 100 × 1 random noise vector, molded with a 14 × 14 × 512 matrix, and fed into the dense layer. In this architecture, one conv2D layer and four convolution2D transposers are used, resulting in images with sizes from 14 × 14 × 512 to 224 × 224 × 3. The first Convolutional2D transpose layer reshapes the image size from 14 × 14 × 512 to 28 × 28 × 256. The architecture is the similar for rest of the layers. The output is reshaped to 56 × 56 × 128, 112 × 112 × 64, and 224 × 224 × 32 through the Conv2D transpose layer, the activation function LeakyReLu, and the batch normalization layer, respectively. Thus, we get an image of size 224 × 224 × 3 as a result.

The images of both the genuine dataset and the artificially generated dataset are presented as input to the discriminator. This has four convolution blocks, each of them containing a dropout layer and an activation function, LeakyReLu. The discriminator which is a binary classifier operator predicts whether an image is false or real when the input image passes through the four blocks. The discriminator mistakes the generated sample image for a real image only if the real and generated images are close. The discriminator can identify fake images when the images are not similar to the real ones. This helps to acquire the gradient which updates the weights through backpropagation. As the weights are updated, a more robust generator is built through training. This is how better fake images to trick the discriminator are produced.

**Fig. 5:**
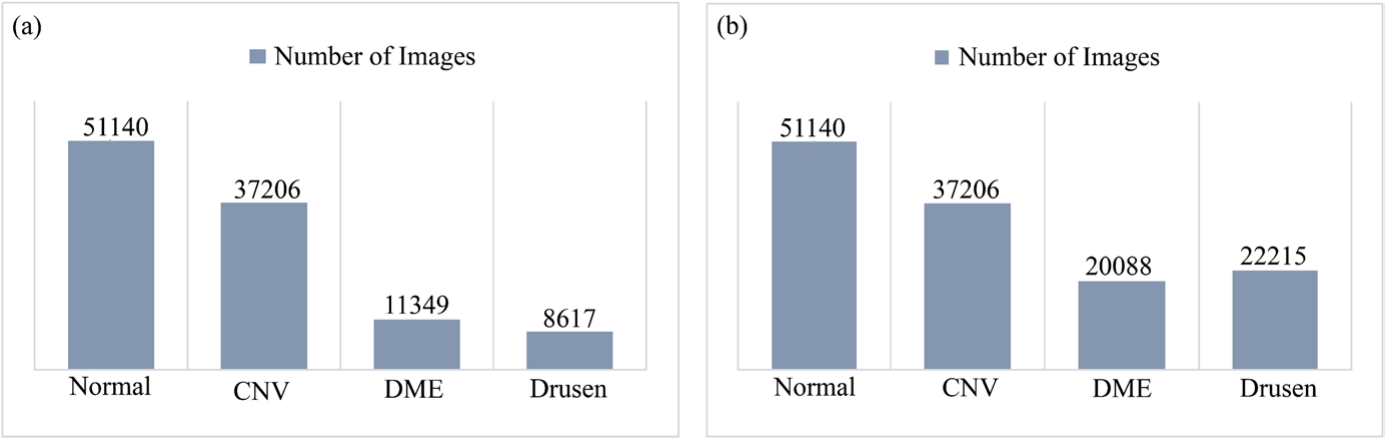
Comparison of class distribution. **a** The skewness of the classes before GAN. **b** The skewness of the classes after GAN

The dataset contains four classes which are not balanced. The normal class includes 51,140 images. This is the class with the highest number of images. The second largest class is CNV, containing 37,206 images. The other two classes (DME & Drusen) are augmented by generating images, resulting in a number that is quite close to the second-highest class, CNV. The images in our dataset are resized into 224 × 224 before applying DCGAN. In training the DCGAN model, the following parameters were employed: the Adam optimizer, a learning rate of 0.0008, a batch size of 128, and binary cross-entropy as the loss function. The number of epochs is chosen based on the image numbers in the original dataset. For the DME class, 400 epochs are used for model training because the initial set of Images (11,349) contains enough different images for these epochs. On the other hand, for the Drusen class, the epoch number was 250 because this class had fewer images (8617). To present the model’s generalization capability, we intentionally generated 8739 images for the DME class and 13,598 images for the Drusen class, resulting in approximately 20 k samples for each. We chose to synthesize around 20 k images for the DME and Drusen classes instead of 30 k (close to CNV class) to prevent over-representation of minority classes. While balancing datasets is a common practice to prevent models from becoming biased towards overrepresented classes, creating an exactly equal number of images with GAN for each class can introduce issues. Though GANs can generate realistic data, they are prone to mode collapse, when the diversity of generated samples is low. This means GANs might bias the synthetic data towards majority demographics or common image pattern, potentially missing the presence of lower number of images with uncommon patterns [23]. Generating a vast amount of synthetic data to balance the dataset, especially when the original data distribution is significantly skewed (see Fig. 5a), it can compromise model reliability. For example, in our dataset, compared to the CNV and normal classes, the DME and Drusen classes lack a significantly high amount of data. Creating synthetic data at this scale may lead to the model overly relying on artificial data, which might not accurately work for real-world conditions [23]. This reliance can result in issues like underfitting, where the noise introduced by synthetic samples prevents the model from capturing the true underlying patterns in the data [24], or overfitting, where the model performs well on synthetic data but poorly on real-world data [25]. Moreover, we have not produced an exactly equal number of images for each class, because, with such a balanced dataset, the model’s effectiveness cannot be assessed.

The utilization of DCGAN resulted in an expansion of the dataset from 108,312 images to 130,649 images. Table 2 presents the total count of images generated with DCGAN, along with the original number of images.

**Table 2:** The original and DCGAN generated image

Classes	Number of images in original dataset	Training images for DCGAN	Generated images By DCGAN	Total
CNV	37,206	-	-	37,206
DME	11,349	2000	8739	20,088
Drusen	8617	2000	13,598	22,215
Normal	51,140	-	-	51,140
	Total = 108,312			Total = 130,649

**Fig. 6:**
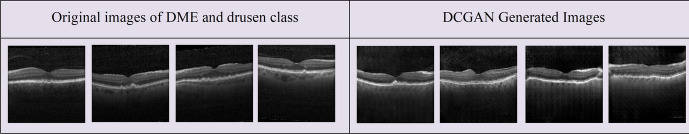
Original images with DCGAN generated images

Figure 6 shows original images of the DME and Drusen classes from the Mendeley OCT dataset as well as DCGAN generated images for each of the two classes. It demonstrates how well our DCGAN model performs by generating the smooth and highly defined layers of retinal OCT images. The images of the original classes and the DCGAN generated classes are hardly distinguishable from each other.

### Image Preprocessing

Image preprocessing is a crucial step which leads to better model accuracy, robustness, efficiency, and time complexity by ensuring that the model is processing information consistently across all images. Generally, OCT images contain speckle noise. Also, the images of the dataset contain white borders and several artifacts. The artifacts are removed by applying morphological erosion. Subsequently, a median filter is used in order to smooth the image and remove the speckle noise. Finally, alpha–beta correction is applied to adjust the brightness and contrast level.

### Morphological Erosion

Morphological erosion computes a local minimum of a given kernel which is used to remove an object’s boundary pixels. In this operation, noise and artifacts of the input image are removed using a structuring element. The object’s boundary pixels elimination depends on the size and shape of the kernel. The algorithm works using the following formula [26].

2 $$f \theta S= {min}_{S}(f(m, n)\cap {S}_{mn}$$

where $f(m, n)$ denotes the input grayscale image and ${S}_{mn}$ is the reference point of the structuring element *S* which is present at the image coordinates *m*, *n*. The min function computes the minimum value of all the pixels under the structuring element, and at position (*m*,* n*), we get the output pixel. In this study, applying a kernel size of 5 × 5, the operation effectively removes artifacts and produces a noise free image.

### Median

The median filter is a digital signal processing filter that works by replacing each pixel in the picture with the median value of its neighbors, in order to minimize the noise in the image. It preserves sharp edges and is an efficient way to smooth spiky noise. It moves pixel by pixel through the image and replaces each pixel value with the median pixel value of the window [27]. This is an effective and simple yet powerful method to remove image noise.

### Alpha–Beta Correction

Alpha–beta correction is a data filter where the alpha is used for contrast control and the beta is used for brightness control [28]. For lower contrast, the alpha value can be set between 0 and 1. For higher contrast, the alpha value should be larger than 1. The range of beta is [− 127, 127]. The output of every pixel is contingent on the value of the corresponding input pixel.

3 $$p\left(x\right)=af\left(x\right)+\beta$$

where *α* and *β* are gain and bias parameters, respectively. The source image pixel is denoted by *f(x)* and *p(x)* is the output image pixel.

**Fig. 7:**
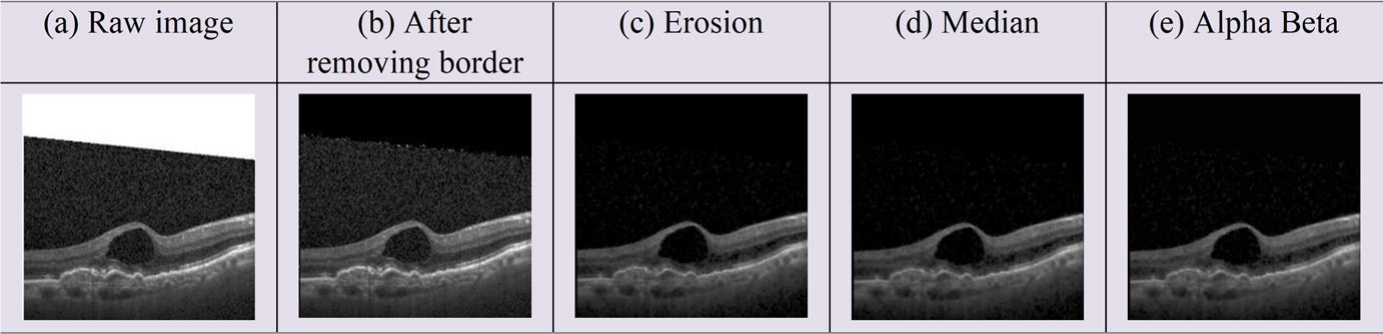
Image pre-processing steps

In this study, we have used the alpha value of 1 and the beta value of 2. Figure 7 illustrates the output image after every image preprocessing step.

Figure 7 shows sample images after each processing step. (a) The original images contain white borders and speckle noise. (b) The white borders are removed. (c) In order to eliminate artifacts and mitigate speckle noise within the images, morphological erosion is implemented. (d) Median filter is applied to get a clearer view of the image, and the speckle noise is successfully eliminated. (e) Alpha–beta correction is applied to highlight the retinal layers.

### Model

To conduct the classification, a vision transformer-based model called CCT is fine-tuned through ablation study. The proposed optimized CCT (OCCT) model combines a lightweight architecture with higher accuracy. The ViT and Swin models are also employed and compared with the proposed OCCT model. The ViT and Swin Transformer models are trained using the same optimizer, activation function, loss function, pooling layer, and learning rate as the OCCT model. This section provides a comprehensive explanation of the models.

### Proposed OCCT Model

CCT model combines the advantages of transformers and convolution. CCT is the latest version of ViT and is regarded as a data-hungry model. Transformer with sequential pooling and convolutional tokenization are the major building blocks of CCT architectures. Compared to the ViT, the convolutional tokenizer is more efficient in CCT. A series of convolutional layers, an input layer, and a self-attention mechanism make up the basic structure of the CCT model, providing computational efficiency. These modules enable the model to concentrate on pertinent features and their relationships.

### Convolutional Tokenization

The convolutional tokenization block is used to identify images by passing their patches through a transformer encoder. The transformer encoder features dropout, ReLU activation, and layer normalization (LN) which is applied after learnable positional embedding. The augmented images are sent to an input layer with dimensions of 32 × 32 × 3. The following equation can be used to represent the convolutional tokenization module.

4 $$\text{X}0 =\text{ MaxPool}(\text{ReLU}(\text{Conv}2\text{D}(\text{x})))$$

Here, X0 represents the input image, the convolutional 2D operation has 64 filters with the ReLU activation function, and MaxPool represents the pooling layer. The convolutional block allows the model to construct a feature map and retain local spatial information. Image patches from this block which are then sent to the transformer-based backbone.

### Transformer Encoder

The transformer encoder is a critical component of the CCT model’s design, responsible for capturing the correlations between various image components. It is composed of a sequence of layers, with each layer featuring spatial attention and a position-wise fully connected feed-forward neural network. Non-linear interactions between distinct characteristics are possible with the position-wise feed-forward neural network. The encoder block, which consists of a self-attention layer and a multilayer perceptron (MLP) head, subsequently receives the output image. The transformer encoder employs dropout, ReLU activation, and layer normalization (after positional embedding).

### Sequential Pooling

In order to map sequential outputs and weigh the latent spaces sequential embeddings, sequence pooling is used. Sequential layers pool the sequence of embeddings and comprise the data into relevant information. The network uses sequence pooling to weigh the sequential embeddings of latent spaces. Then sequence pooling layer pools the full sequence of data since it contains pertinent data from different input image sections. This operation can be described as

5 $${y}_{L}=f\left({y}_{0}\right)\in {H}^{(s\times g\times e)}$$

where *L* is a layer transformer encoder whose output is ${y}_{L}$. The mini-batch size is denoted by *s*, the embedding dimension is denoted by* e* and the sequence length is denoted by *g*. The softmax activation function (Eq. 7) is then applied to a linear layer called $j({y}_{L})$. After pooling the second dimension, the result is $z\in {H}^{\left(s\times e\right)}.$ Then, the images are categorized. The output operation can be given as (Eq. 8).

6 $${y}_{L}{\prime}=softmax(j({y}_{L}{)}^{T})\in {H}^{(s\times 1\times g)}$$

7 $$z={y}_{L}{\prime}{y}_{L}=softmax(j({y}_{L}{)}^{T}){\times y}_{L}\in {H}^{\left(s\times 1\times e\right)}$$

**Fig. 8:**
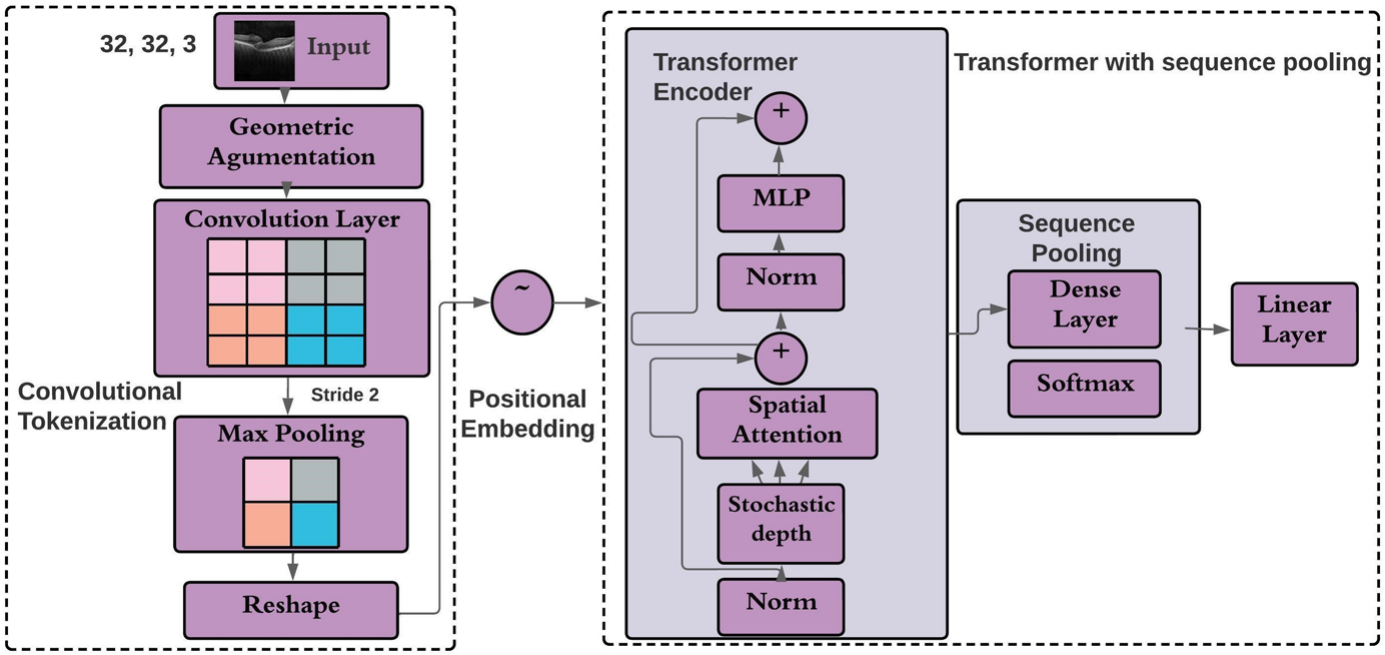
CCT model’s key modules

Figure 8 describes the modules and layers that comprise the CCT architecture.

The following is a step-by-step breakdown of the base CCT architecture:

- Geometric augmentations are performed to the input image.
- The mages are transferred to the CCT convolution layer.
- With the help of convolution and max pooling, the output image is reshaped.
- Before being directed towards the transformer encoder, the resulting image data is handled by TensorFlow extensions.
- The transformer encoder block performs layer normalization, followed by spatial attention and regularization.
- Similar to the preceding transformer encoder, two more transformer encoder employs a regularization layer to further regularize the output.
- As a result, the first steps in the transformer encoder comprise layer normalization, two pairs of dense and dropout layers, and regularization, all of which are performed using TensorFlow addons.
- The normalized output is sent via a dense and a SoftMax layer, which form the final output.

**Fig. 9:**
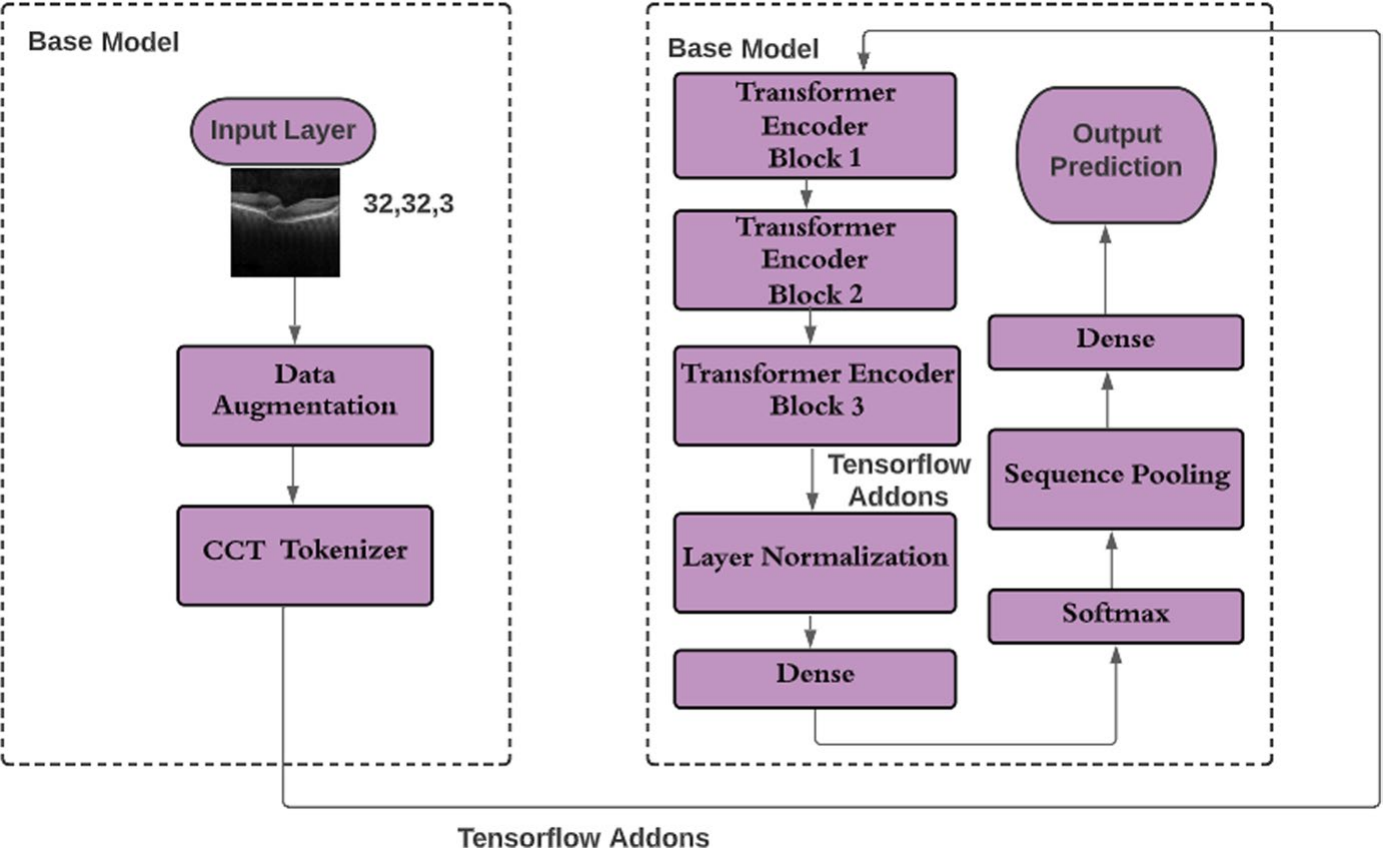
CCT base model architecture

This work proposes the OCCT through ablation research to the core CCT model. The base architecture of CCT is shown in Fig. 9.

The general architecture of the compact convolutional transformer model is as follows:

- In the data augmentation part, the model applies the usual geometric augmentations to input images.
- The images are passed to the CCT tokenizer, where the output image is reshaped using the convolution tokenizer and max pooling. Here, the stride and kernel sizes for the CCT tokenizer block’s convolution layer are 2 and 4, respectively.
- The set of transformer blocks that come after these convolutional layers uses self-attention methods in order to enhance the features and identify long-range dependencies between them.
- Before sending the output image data to the transformer encoder block, TensorFlow extensions are utilized to process it. The layers in this block are categorized into two groups using two pairs of dense and dropout layers: layer normalization is applied in the first group, while spatial attention regularization is implemented in the second group. Another regularization layer is added to the last layer of the transformer encoder block. The regularization layer’s output was then routed to the layer normalization.
- The CCT model uses layer normalization to increase the stability and effectiveness of the training process. The activations of the neurons inside a layer are normalized by normalization layers, which can help the model converge more rapidly during training by preventing activations from being excessively large or tiny. The normalized output is subjected to a dense and SoftMax layer to generate the final output.
- After the last dense layer produces a vector of logits, the SoftMax activation function is applied, followed by a SoftMax layer, to determine the final output probabilities in the CCT model. Sequence pooling layers create a single feature vector that represents the whole input sequence, which can assist in reducing the input’s dimensionality and enhancing the model’s effectiveness.
- Ultimately, the CCT model’s dense, SoftMax, and sequence pooling layers collaborate to construct the final output classification probabilities from the input image. The dense layers modify the retrieved features linearly, the SoftMax layer normalizes the output probabilities, and the sequence pooling layer collects the features over many spatial locations.

### Ablation Study

An ablation study on the core CCT model is conducted to optimize the performance. This involves changing the type of activation functions and pooling layers, altering the number of transformer encoders, and experimenting with different pooling layers, stride sizes, kernel sizes, loss functions, optimizers, learning rates, and batch sizes. After completing all the ablation experiments, the OCCT model results in better accuracy and faster performance. The purpose of the ablation study is to analyze the effect of various components on the model’s performance. The section 4.2 contains the results of ablation study.

### Proposed OCCT Model

**Fig. 10:**
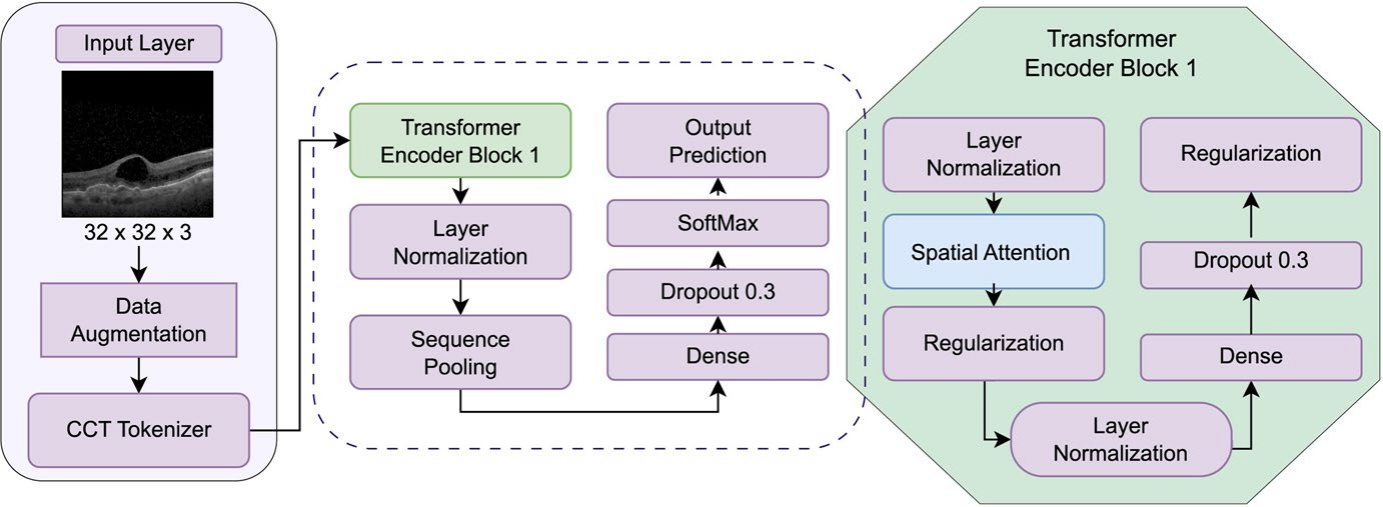
Proposed OCCT model architecture

The resulting OCCT architecture, demonstrated in Fig. 10, is similar to the base CCT and has only one transformer encoder block rather than three, simplifying the model and allowing for faster training. Compared to the proposed model, the OCCT model has shorter training and testing times and requires fewer resources, as it does not include positional encoding and relies solely on the self-attention technique in the transformer structure. The remaining components of the architecture are the same with some modifications made to the model hyperparameters, including the kernel size and stride size. As a result, the OCCT model is more efficient.

### Comparison with Two Transformer Models

The performance of OCCT is compared with a vision transformer and a Swin Transformer. This section provides a detailed description of these models. The OCCT model is a hybrid architecture that combines the advantages of self-attentional mechanisms and convolutional neural networks (CNNs). The OCCT model utilizes a CNN to extract local features from the input image and self-attention mechanisms to model long-range relationships between these features. As a result, the OCCT model can gather both local and global context information. In contrast, the Swin Transformer model is a hierarchical architecture that combines the advantages of self-attention and CNNs, and the ViT model is exclusively based on self-attention processes.

### Vision Transformer

**Fig. 11:**
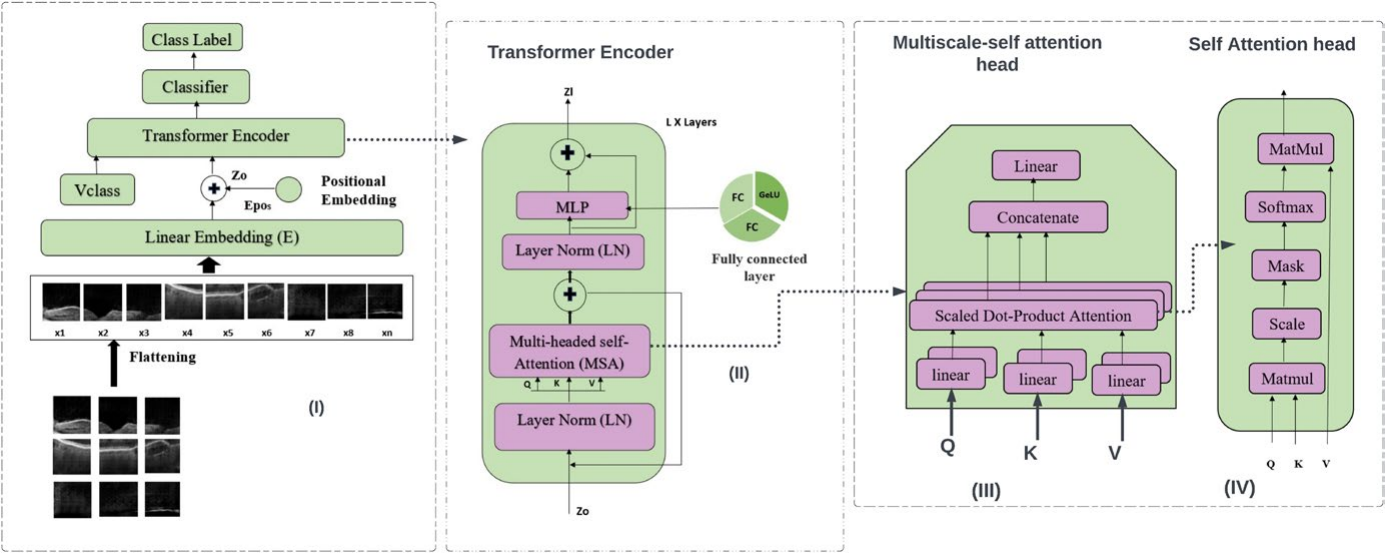
(I) The vision transformer architecture with the (II) transformer encoder component, (III) head as multiscale-self attention (MSA), and (IV) also the self-attention (SA) head

ViT is a graphical representation of a transformer’s architecture. The model was introduced by [29]. An input image is represented by the ViT model as a collection of image patches. The visual transformer can modify an image by segmenting it into fixed-size patches, precisely embedding each patch, and including positional embedding as input to the transformer encoder to model long-range dependencies between features. In ViT, there are various features: positional embedding, image tokenization, transformer encoder, classification token, and classification head. The core architecture of a vision transformer is shown in Fig. 11.

ViT breaks an image into non-overlapping square patches and arranges them linearly. The learnable positional embedding, which concatenates the patch embedding, stores the spatial location of each patch. The total number of pixels in the image and the size of the patches are used to calculate the number of patches. Two-dimensional images can be divided into smaller, non-overlapping patches for processing. Let *H* and *W* represent the height and width of the image, respectively. If each patch has dimensions *P* × *P*, then the total number of patches, *N* is given by $N= \frac{H\times W}{{P}^{2}}$ In this equation, *H* and *W* denote the image dimensions, *P* is the side length of each square patch, and ${P}^{2}$ represents the area of each patch. Thus, *N* represents the total number of such patches covering the entire image [30]. To create the token embeddings, the sequence of patches is flattened into a 1D vector using a linear layer. The linear embedding layer includes two linear transformations: the patch projection, which maps each patch embedding to a higher-dimensional vector space, and the positional encoding projection, which provides information about the spatial placement of each patch in the input image. The sequence of patches is mapped to a model vector using a learned embedding matrix *E*. The transformer sees the inserted picture patches as a collection of patches with no understanding of their link to one another. The positional information is encoded as Epos and added to the patch representations to maintain the spatial arrangement of the patches in the original image. The sequence of embedded patches with the token z0, along with the positional encoding, can be expressed as shown in Eq. 9.

8 $${z}_{0}=\left[{v}_{class};{x}_{1}E;{x}_{2}E;\dots ;{x}_{n}E\right]+{E}_{pos},E\in {R}^{\left({p}^{2}c\right)\times d},{E}_{pos}\in {R}^{\left(n+1\right)\times d}$$

We employ traditional learnable 1D position embeddings. The created sequence of embedding vectors is the input of the encoder. The latent data that will subsequently be used for classification are contained in the [class] token. The transformer encoder consists of several identical blocks, each with two sub-layers: a multi-head attention layer and feedforward layer. It also has a multi-head self-attention block (Eq. 10). The model can represent long-term interdependence and spatial interactions between patches, thanks to the multi-head self-attention layer. It performs operations on the input vector sequence by computing self-attention scores between all pairs of vectors in the sequence. The feed-forward layer performs a non-linear transformation to the output of MSA (multi-headed self-attention). It consists of two linear transformations, separated by the GELU activation function (Eq. 11). To increase the model’s stability and convergence, each transformer encoder block incorporates residual connections and layer normalization (Eq. 12). The residual connection allows the output of a block to be added to its input, while the layer normalizes the output of the block to have zero mean and unit variance. The various attention heads aid in the training of an image’s regional and global dependencies.

9 $${z}_{l}{\prime}=MSA\left(LN\left({Z}_{l-1}\right)\right)+{Z}_{l-1},l=1\dots L$$

10 $${\text{z}}_{l}=MLP\left(LN\left({\text{z}}_{l}{\prime}\right)\right)+{\text{z}}_{l}{\prime},l=1\dots L$$

11 $$yy=LN\left({Z}_{l}^{0}\right)$$

The ViT model’s input features are subjected to a self-attention operation by the MSA block, allowing it to recognize the dependencies and connections between various elements of the input image. The location of the picture representation is determined by the position of the [class] token. A “transformer” computational intelligence model uses selective attention mechanisms to evaluate the significance of each portion of the input data separately. The self-attention technique’s ability to discern long-term correlations between sequence components has had a substantial impact on transformer model accuracy.

### Swin Transformer

**Fig. 12:**
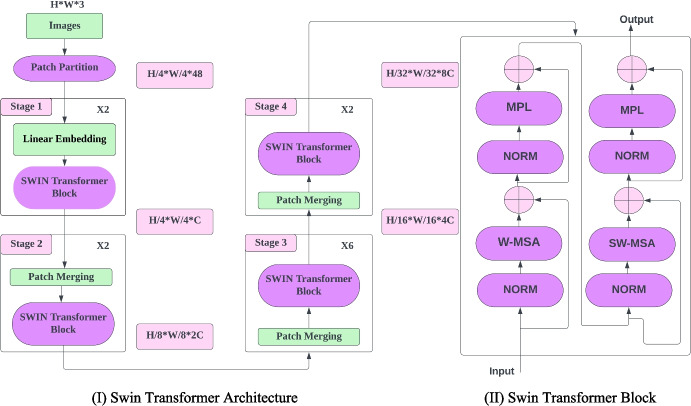
(I) The architecture of a Swin Transformer and (II) Swin Transformer Block with 2 sub-units. The first sub-unit applies W-MSA and the second sub-unit applies SW-MSA, multi-head self-attention modules that can be configured with conventional or shifted windowing

The Swin Transformer overcomes the shortcomings of the ViT by creating hierarchical feature maps and shifting windows. For MLP architectures, the hierarchical design and the shifted window technique work well [31]. Figure 12 gives an overview of the Swin architecture.

The term “hierarchical” in Swin Transformer refers to the process of merging feature maps from one layer to another, which results in a reduction of the spatial dimension of the feature maps. This is achieved through patch merging, which involves the gradual merging and down sampling of feature maps. Patch merging concatenates features of each set of *n* × *n* neighboring patches, resulting in a down sampling of feature maps by a factor of *n*. Depth-wise grouping and combination of *n* × *n* neighboring patches are performed to create hierarchical feature maps [31]. The Shifted Window MSA (SW-MSA) and Window MSA (W-MSA) modules replace the ViT’s multi-head self-attention (MSA) module in the Swin Transformer. Each module includes an attention module, an MLP layer, a normalization layer, and a further normalization layer.

The Swin Transformer utilizes a Window-based MSA module that performs attention computation within each window of 2 by 2 patches. Since the window size is fixed, the complexity of the window-based MSA is linear with respect to the number of patches. However, its limitation is that it only allows self-attention within each window. To overcome this, the Swin Transformer introduces the Shifted Window MSA (SW-MSA) module, which enables cross-window connectivity. The SW-MSA module is used after the W-MSA module in the Swin Transformer architecture. Swin Transformer’s “Cyclic Shift” approach inserts “abandoned” patches into windows. Because a window can now comprise patches, a filter is utilized during the calculation to limit self-attention to neighboring patches. This approach of window shifting introduces connections across windows and has been shown to improve network efficiency [31].

### Comparison with Transfer Learning Models

Eight transfer models, including DenseNet121 [32], DenseNet201 [33], ResNet50 [34], MobileNetV2 [35], ResNet101V2 [36], VGG16 [37], VGG19 [38], and EfficientNetB1 [39], are implemented in this study and compared with the proposed OCCT model. The models are evaluated with the same configurations as the OCCT model, where we have used a batch size of 128, “Adam” optimizer, “categorical crossentropy” as the loss function, a learning rate of 0.001, and the activation function “elu.” Each model has a unique architecture and a unique method for making predictions. DenseNet is an image classification algorithm that was created to address the issue of vanishing gradients and increase the accuracy of a model. By simply connecting every layer directly to one another, DenseNet eliminates this gradient problem resulting in improved accuracy. DenseNet201 transition layers assist in lowering the network’s computational expense while preserving accuracy. ResNet-50 has demonstrated cutting-edge performance in image classification, object detection, and semantic segmentation. MobileNetV2 is a shallow network, trained with the ImageNet dataset, a sizable dataset with 1.4 million images and 1000 classes. ResNet101V2 uses residual blocks to avoid the vanishing gradient problem by allowing gradients to flow straight through the network. It also skips connections and batch normalization layers to increase network stability and convergence. The VGG-16 architecture was specifically developed for image classification tasks and has achieved outstanding performance on the ImageNet dataset. VGG-19 is a well-known architecture in computer vision research that has inspired numerous subsequent convolutional neural network implementations. EfficientNetB1 adopts a compound scaling approach that resizes the network’s depth, width, and resolution in a systematic way, which improves its efficiency and accuracy. This enables the development of a family of models that outperform earlier architectures while being smaller and faster.

### Computing Environment

The experiments are carried out on a computer with an Intel Core i5-8400 processor and 16 GB of installed RAM. An NVIDIA GeForce GTX 1660 GPU boosts the processing power to effectively handle deep learning workloads. A fast 256 GB DDR4 SSD fulfills the need for storage.

## Results and Discussion

In this section, the results of this overall study, including the ablation studies, transformer, and transfer learning model evaluation, and model evaluation matrix are discussed. Additionally, a discussion is offered based on the limitations.

### Evaluation Metrics

In classification tasks, the results are evaluated based on the true positive (TP), true negative (TN), false positive (FP), and false negative (FN) values obtained from the confusion matrix [36].

### Result of the Ablation Study

This section presents the results of the ablation study, which explores different hyperparameters and layer architectures of the proposed model to improve its performance and reduce computation time. Using an epoch size of 100, we have done seven experiments, shown in Table 3, altering different components of the OCCT model, to achieve a more reliable architecture with improved classification accuracy.

**Table 3:** Ablation study

Study 1: Changing the transformer layer					
Configuration number	Transformer encoder block number	Number of parameter	Training time	Accuracy	Findings
**1**	**1**	**0.24 M**	**19 s**	**91.25%**	**Close to highest accuracy with lowest computational time**
2	2	0.41 M	38 s	91.41%	Moderate time with close highest accuracy
3	3	0.57 M	60 s	91.53%	Highest accuracy with highest computational time
Study 2: Changing the kernel size					
Configuration number	Kernel size	Parameter number	Training time	Accuracy	Findings
1	1	0.17 M	23 s	87.30%	Close to high accuracy
2	2	0.2 M	24 s	92.12%	Close highest accuracy
**3**	**3**	**0.24 M**	**19 s**	**93.57%**	**Highest accuracy with lowest complexity**
4	4	0.3 M	21 s	92.31%	Lowest accuracy
5	5	0.37 M	25 s	92.82%	Lower accuracy
Study 3: Changing the activation function					
Configuration number	Activation function	Training time		Accuracy	Findings
1	relu	19 s		93.57%	Close to highest accuracy
**2**	**elu**	**19 s**		**93.82%**	**Highest accuracy**
3	Tanh	19 s		93.37%	Close to highest accuracy
4	softplus	19 s		91.72%	Lower accuracy
5	softsign	19 s		92.37%	Lower accuracy
6	LeakyReLU	19 s		91.25%	Lower accuracy
7	GELU	19 s		92.84%	Lowest accuracy
8	sigmoid	19 s		91.20%	Lower accuracy
Study 4: Changing the pooling layers					
Configuration number	Pooling layer	Training time		Accuracy	Findings
1	**Max**	**19 s**		**93.82%**	**Highest accuracy**
**2**	Average	19 s		92.55%	Lowest accuracy
Study 5: Changing the optimizer					
Configuration number	Optimizer	Training time		Accuracy	Findings
**1**	**Adam**	**19 s**		**95.48%**	**Highest accuracy**
2	Adamax	19 s		94.17%	Accuracy improved
3	Nadam	19 s		93.82%	Lower accuracy
4	SGD	19 s		86.18%	Lowest accuracy
5	RMSprop	19 s		95.04%	Accuracy improved
Study 6: Changing the learning rate					
Configuration number	Learning rate	Training time		Accuracy	Findings
1	0.01	19 s		84.06%	Lowest accuracy
**2**	**0.001**	**19 s**		**97.09%**	**Highest accuracy**
3	0.006	19 s		89.3%	Lower accuracy
4	0.0008	19 s		95.19%	Close highest accuracy
Study 7: Changing the loss function					
Configuration number	Loss function	Training time		Accuracy	Findings
**1**	**Categorical Crossentropy**	**19 s**		**97.09%**	**Highest accuracy**
2	Binary Crossentropy	19 s		95.23%	Close to highest accuracy
3	Mean squared error	19 s		95.39%	Close to highest accuracy
4	Mean squared logarithmic error	19 s		15.32%	Lowest accuracy
5	Mean absolute error	19 s		92.31%	Lower accuracy

**Fig. 13:**
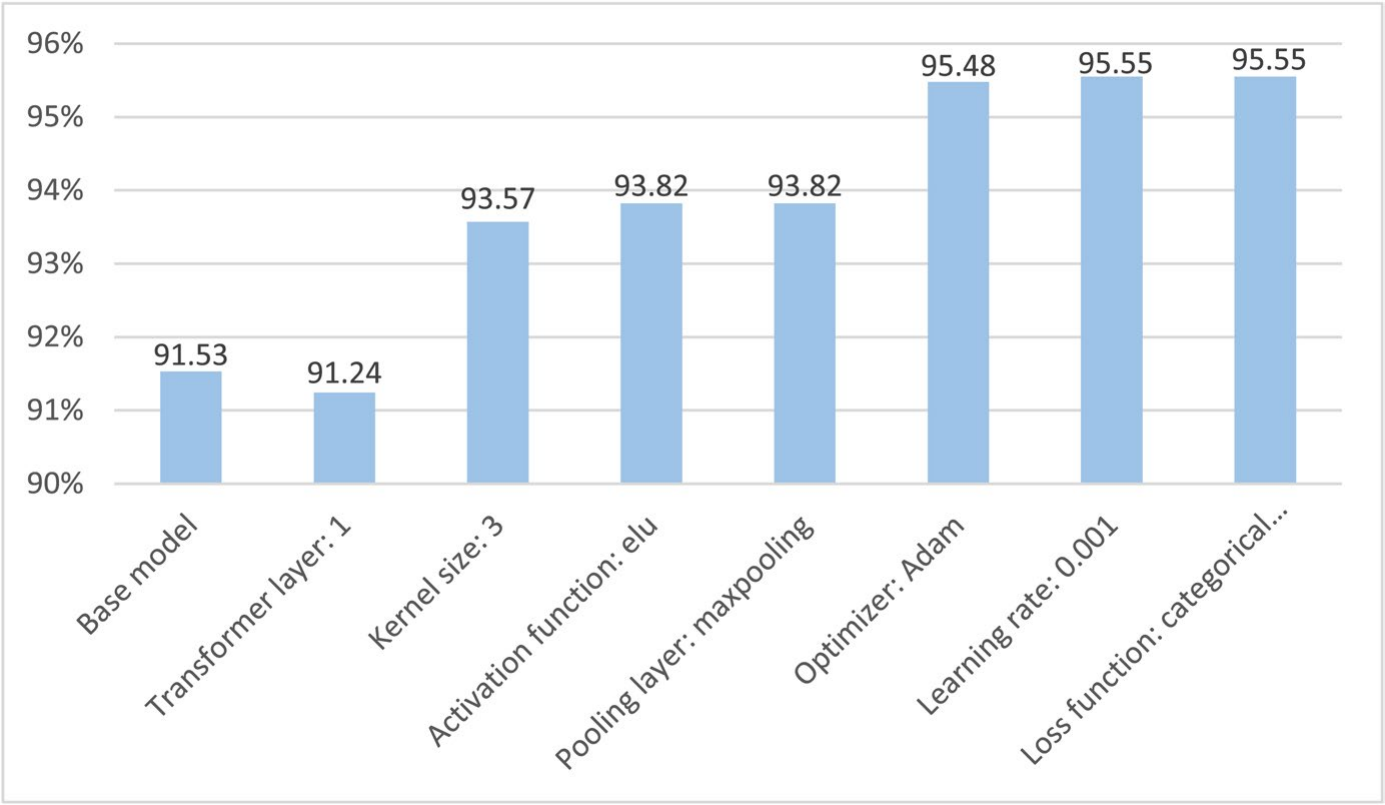
The improved configuration of the OCCT model is demonstrated

This study focuses on optimizing the model’s performance while maintaining computational efficiency. Therefore, the selected range of hyperparameters for each experiment was chosen based on balancing accuracy improvement and resource consumption. In study 1, experiments are done with different numbers of transformer encoder blocks. While increasing the number of transformer blocks from 1 to 3, it is observed that the accuracy increases marginally with each additional block. Specifically, the accuracies for 1, 2, and 3 transformer blocks were 91.25%, 91.41%, and 91.53%, respectively. However, the number of parameters and the training time increased significantly with the number of transformer blocks. The parameter counts for configurations with 1, 2, and 3 blocks were 0.24 M, 0.41 M, and 0.57 M, respectively, and the corresponding training times were 19 s, 38 s, and 60 s per epoch. This indicates that while the accuracy difference between the configurations is minimal (only a 0.28% increase from 1 to 3 blocks), the computational cost increases substantially, with training times nearly tripling. Due to this, the configuration with 1 transformer block was selected for further experiments offering a good balance between accuracy and computational efficiency. Further experiments were therefore performed with one transformer encoder block. In study 2, the experiment has been run with different kernel sizes of 1, 2, 3, 4, and 5. The optimal accuracy of 93.57% was achieved with a kernel size of 3, which also maintained the same parameter count and training time as for transformer block 1. From kernel size 1 to 3 the accuracy increased substantially. In contrast, when we tested kernel size 4, the accuracy dropped to 92.31%, and both the parameter count (0.3 M) and training time (21 s) increased. The performance of the model with a kernel size of 5 was also tested, but the results showed no further accuracy improvement and a continued increase in computational cost. Therefore, the kernel size range was limited to 1 to 5, identifying size 3 as the most efficient in balancing accuracy and computational demands. So, further evaluation will be done with a kernel size of 3. In the subsequent experimental studies (studies 3, 4, 5, 6, and 7), the number of parameters and training time remained consistent across configurations. Consequently, the configurations were solely selected based on achieving the highest accuracies. We have used eight different activation functions, namely ReLU, exponential linear units (ELU), Tanh, Softplus, Softsign, LeakyReLU, GELU, and sigmoid, to see the impact on the CCT classification model in study 3, and for the further ablation study, elu is chosen as it demonstrates the best accuracy of 95.42%. Two types of pooling layers have been utilized in study 4’s experiment (average pooling and max pooling). The max pooling resulted in an accuracy of 93.82%, and this will be used for further studies. The choice of optimizer can significantly affect the model’s performance. For this reason, the performance of our model has been evaluated using five different optimizers: Adam, Adamax, Nadam, SGD, and RMSprop in study 5. The Adam optimizer results in the highest accuracy of 95.48%. Further experiments are done by changing the learning rate (0.01, 0.001, 0.006, 0.0008) in study 6 where a learning rate of 0.001 shows the highest accuracy, whereas the other learning rates reduce the accuracy percentage. Lastly, in study 7, five different loss functions have been tried, namely, categorical cross entropy, binary cross entropy, mean squared error, and mean squared logarithmic error mean absolute error. Among the loss functions, categorical cross-entropy resulted in the highest accuracy of 97.09%. Figure 13 shows a visualization of the proposed model’s improvements after each ablation study.

We can see that after taking only one transformer layer the overall accuracy decreases slightly to 91.24% compared to the base model’s accuracy of 91.53% on the enhanced preprocessed data. The accuracy improves in the next study where a kernel size of 3 gives an accuracy of 93.57%. Using the activation function elu and a max pooling layer increased the accuracy to 93.82%. The optimizer Adam improves the accuracy to 95.48%. With a learning rate 0.001 and categorical cross-entropy as the loss function, the highest accuracy is 97.09% for our proposed OCCT model. The modified configuration of the proposed model is shown in Table 4.

**Table 4:** The proposed OCCT model’s configuration after the ablation study

Configuration	Value
Epochs	100
Transformer layer	1
Kernel size	3
Parameter number	0.24 M
Activation function	elu
Pooling layer	maxpooling
Optimizer	Adam
Loss function	categorical crossentropy
Learning rate	0.001

### Confusion Matrices of the Proposed Model and Two Transformer Models

**Fig. 14:**
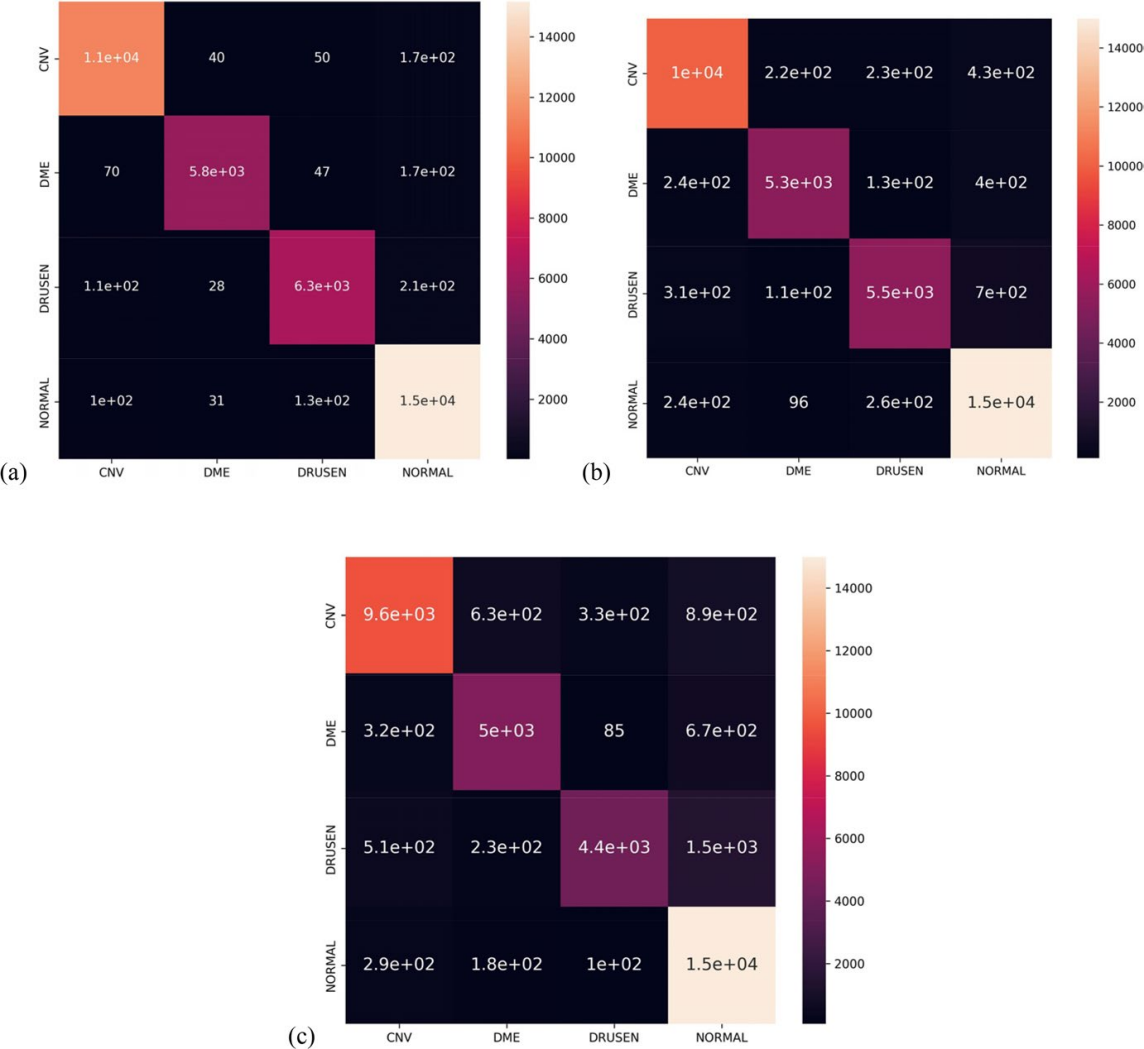
Confusion matrices of proposed **a** OCCT model, **b** Swin model, and **c** ViT model

Figure 14 shows the confusion matrix generated for the OCCT, Swin, and ViT models. The confusion matrices have rows that correspond to the true labels and columns that correspond to the predicted labels of the test images.

In Fig. 14, the confusion matrices for the proposed OCCT model, Swin Transformer, and ViT are displayed. The rows of each matrix indicate the true labels of the test images, and the columns represent the predicted labels generated by each model. The values located on the diagonal of each matrix correspond to the true positive (TP) values. The observations show that our proposed OCCT model does not predict any of the classes better than others and is not biased to any class. However, the other two models (Swin and ViT) seem to be biased on one or multiple classes. In classification tasks, the results are evaluated based on the TP, true negative (TN), false positive (FP), and false negative (FN) values obtained from the confusion matrix.

### Performance Analysis of the Models

The statistical analysis along with the evaluation metrics for the proposed OCCT model and the ViT and Swin model that have been used for the performance comparison are shown in Table 5. We have utilized a variety of metrics, such as precision (Pre), recall, F1 score (F1), specificity (Spe), sensitivity (Sen), NPV, FPR, FDR, FNR, Matthews correlation coefficient (MCC), and the accuracy of the models.

**Table 5:** Performance evaluation of OCCT, Swin, and ViT models

Model	F1 (%)	Pre (%)	Recall (%)	Spe (%)	Sen (%)	NPV (%)	FPR (%)	FNR (%)	FDR (%)	MCC (%)	Accuracy (%)
OCCT	**96.87**	**96.52**	**97.27**	**99.02**	**97.48**	**98.94**	**0.98**	**2.77**	**3.48**	**95.85**	**97.09**
Swin	90.28	89.61	91.44	97.14	91.47	96.89	2.9	8.5	10.3	87.54	91.39
ViT	83.64	82.19	86.24	95.35	86.24	94.69	4.7	13.8	17.8	79.08	85.57

The performance of the proposed OCCT model increased significantly after the ablation study. While evaluating the OCCT model, it performed well compared to the other transformer models. The model achieves an F1 score of 96.87%, whereas the F1 score for ViT and Swin transformer models are 83.64% and 90.28%, respectively. The precision, recall, and specificity for the OCCT model are 96.52%, 97.27%, and 99.02%, respectively, while the precision, recall, and specificity for ViT are 82.19%, 86.24%, and 95.35% and for the Swin model 89.61%, 91.44%, and 97.14%. The sensitivities for the OCCT, Swin transformer and ViT models are 97.48%, 91.47%, and 86.24% respectively. The NPV score for the OCCT model is 98.94%, while the scores for Swin and ViT models are 96.89% and 94.69%. The FPR and FNR for the OCCT model are 0.98% and 2.77%. The FPR and FNR for the Swin model are 2.9% and 8.5% and for the ViT model 4.7% and 13.8%. The OCCT model has the lowest FDR, of 3.48%, whereas the Swin model’s FDR is 10.3% and ViT’s FDR is 17.8%. The highest MCC value of 95.85% is obtained by the proposed OCCT model, while the Swin model and ViT have MCC scores of 87.54% and 79.08% respectively. We can conclude that the OCCT model outperforms the other transformer models.

### The Performance Analysis of Proposed Model on Real-World Data

The classification accuracy of the proposed OCCT model is assessed using dataset that includes GAN-generated synthetic images. In this section, we focus on assessing the model's performance on real-world data to demonstrate its practical effectiveness. To achieve this, the model is trained using an augmented dataset and then tested on a dataset containing only real OCT images. The data distribution used for training and testing the model is detailed in Table 6.

**Table 6:** The distribution of training and testing data

Classes	Number of images in original dataset	Training set (70%)	Testing set (30%)	Training images for DCGAN (taken from training set)	DCGAN generated images	Total training set after GAN
CNV	37,206	26,044	11,162	-	-	26,044
DME	11,349	7944	3405	2000	10,000	17,944
Drusen	8617	6032	2585	2000	12,000	18,032
Normal	51,140	35,798	15,342	-	-	35,798
			Total = 32,494			Total = 65,598

In Table 6, the dataset is divided into 70% for training and 30% for testing. From the training set, 2000 images are used for DCGAN training. Additionally, 10,000 synthetic images are generated for DME and 12,000 for Drusen. The total number of images for model training is 65,598, consisting of augmented data, while 32,494 real images are used for testing the model. The results are shown in Table 7.

**Table 7:** The model’s performance on real image and synthetic image dataset

OCT model’s test data	Accuracy (%)	Pre (%)	Recall (%)	F1 (%)
Tested with augmented dataset	97.09	96.52	97.27	96.87
Tested with real images of the OCT dataset	96.8	96.63	96.25	96.75

Table 7 presents the results for test accuracy, precision, recall, and F1 score for both the real test dataset and the test dataset combined with synthetic images. The accuracy difference between these two is only 0.29%, highlighting the proposed model’s effectiveness. The 30% testing data was kept isolated during model training, ensuring it remained unseen by the model. This demonstrates that the proposed model performed well even on unseen real-world test data.

### Comparison Between OCCT, Swin and ViT Models Based on Decreased Number of Images

This section presents the performance analysis of the OCCT model with decreased amount datasets. The evaluation is carried out 13 times with a reduction of 25% in the dataset each time. As the real retinal OCT dataset is enhanced for the model training, this experiment is done on both real dataset and enhanced dataset. For the experiment, the input image dimension is consistently maintained at 32 × 32 pixels. The learning rate is set to 0.001, the activation function used is elu, the optimizer is Adam, and the batch size is 128.

### Performance of OCCT Model on Enhanced Dataset

The decreasing process for the enhanced dataset results in a total of 4139 images from the original 130,649 images. Table 8 shows the proposed OCCT model, the Swin model and the ViT model’s performance model’s performance for a decreased number of images.

**Table 8:** OCCT, Swin, and ViT model performances on the minimized datasets (enhanced data)

Evaluation no	Number of images	OCCT		Swin		ViT	
		Total time (min)	Test accuracy	Total time (min)	Test accuracy	Total time (min)	Test accuracy
1	130,649	**32**	**97.09%**	65	91.39%	95	85.57%
2	97,987	**25**	**96.34%**	52	91.02%	73	87.31%
3	73,493	**20**	**95.17%**	35	89.88%	58	86.88%
4	55,119	**14**	**94.40%**	25	86.78%	45	85.62%
5	41,338	**12**	**93.70%**	20	86.16%	35	83.25%
6	31,003	**7**	**92.55%**	15	84.36%	25	83.12%
7	23,252	**5**	**91.57%**	12	83.75%	23	79.89%
8	17,439	**6**	**91.91%**	8	79.33%	14	77.18%
9	13,079	**3**	**88.51%**	8	76.85%	15	74.41%
10	9809	**3**	**89.20%**	7	75.35%	8	73.51%
11	7357	**2**	**87.26%**	5	73.11%	7	72.29%
12	5518	**2**	**86.21%**	3	71.58%	5	71.09%
13	4139	**2**	**85.60%**	3	70.54%	3	69.98%

If we compare the performances of the OCCT, Swin Transformer, and ViT models, we can see that the OCCT model performs better than the ViT and Swin transformer models. The OCCT model outperformed the other transformers even for the smallest number of images (4780 images) where OCCT yields an accuracy of 85.60% and the Swin Transformer acquires an accuracy of 70.54%. In contrast, the ViT model only has an 85.57% accuracy with the highest number of images (130,649 images), and it is considered the weakest model for this application. The accuracy drops from 97.09 to 85.60% for the OCCT model. For Swin and ViT models, the accuracy fall is 91.39 to 70.54% and 85.57 to 69.98%, respectively. So, here, we cannot see any drastic accuracy drop for the reduced datasets. For OCCT model, the accuracy drops gradually, and it only drops 10% accuracy, while the data has been reduced by almost 97% (130,649 to 4139 images). For the Swin Transformer models, the accuracy collapses over 20%, and for the ViT model, the accuracy drops over 15%. So, in comparison to OCCT, no model was able to maintain efficiency as well as OCCT has. Despite the limited sample, the OCCT observation is remarkable.

### Performance of OCCT Model on Real Dataset

The reduction process for the real retinal OCT dataset yields a total of 3430 images from the original 108,312 images. Table 9 presents the performance of the proposed OCCT model, along with the Swin and ViT models, on the reduced dataset.

**Table 9:** OCCT, Swin, and ViT model performances on the minimized datasets (real data)

Evaluation no	Number of images	OCCT		Swin		ViT	
		Total Time (min)	Test accuracy	Total time (min)	Test accuracy	Total time (min)	Test accuracy
1	108,312	**27**	**91.49%**	55	87.89%	87	77.23%
2	81,234	**23**	**89.67%**	37	85.99%	61	76.18%
3	60,925	**16**	**86.23%**	29	83.13%	53	74.51%
4	45,694	**13**	**84.63%**	24	81.65%	38	73.29%
5	34,270	**8**	**83.18%**	19	80.75%	28	72.09%
6	25,702	**7**	**81.67%**	14	79.17%	26	69.41%
7	19,277	**6**	**78.29%**	10	75.77%	15	67.20%
8	14,457	**4**	**77.91%**	9	75.37%	14	65.98%
9	10,843	**3**	**75.33%**	7	73.14%	10	63.37%
10	8132	**3**	**74.91%**	7	71.11%	9	63.02%
11	6099	**2**	**73.21%**	4	69.25%	7	61.77%
12	4574	**2**	**71.01%**	2	65.69%	3	59.13%
13	3430	**1.3**	**70.75%**	2	65.37%	2	58.33%

The proposed model achieved an accuracy of 91.49% on the real imbalanced retinal OCT dataset, outperforming the Swin and ViT models. The accuracy range for the OCCT model on reduced datasets was 91.49 to 70.75%, compared to 87.89 to 65.37% for the Swin model and 77.23 to 58.33% for the ViT model. This demonstrates the superior performance of the proposed model, even under challenging conditions.

### Comparison with Transfer Learning Models

Eight transfer learning models were trained with the DCGAN-generated enhanced dataset with an image size of 32 $\times$ 32 pixels, batch size of 128, learning rate of 0.001, activation function elu, and optimizer Adam. Table 10 compares the results of our proposed OCCT model with the transfer learning models. DenseNet121 achieved the highest accuracy of 72.21% and the lowest computational time among the transfer learning models. The other models had accuracies ranging from 67 to 39%. However, despite using a large number of training images, the transfer learning models performed poorly compared to the proposed OCCT model, which achieved an accuracy of 97.09% and a training time of only 32 min, significantly lower than the training time of the transfer models. Therefore, we can conclude that the proposed model outperforms both transformers learning models and transfer learning models.

**Table 10:** Performance compatibility analysis of transfer learning models with the OCCT model

Model	Number of images	Time (s) $\times$ epochs	Total time (min)	Accuracy
DenseNet121	130,649	143 s $\times$ 100	237	72.21%
DenseNet201	130,649	153 s $\times$ 100	255	66.48%
ResNet50	130,649	158 s $\times$ 100	264	55.95%
MobileNetV2	130,649	159 s $\times$ 100	265	53.79%
ResNet101V2	130,649	156 s $\times$ 100	260	53.74%
VGG16	130,649	155 s $\times$ 100	259	50.51%
VGG19	130,649	163 s $\times$ 100	272	41.11%
EfficientNetB1	130,649	143 s $\times$ 100	239	39.22%
**OCCT**	130,649	**19 s ** $\times$** 100**	**32**	**97.09%**

### Performance Evaluation Using K-Fold Cross-Validation

**Fig. 15:**
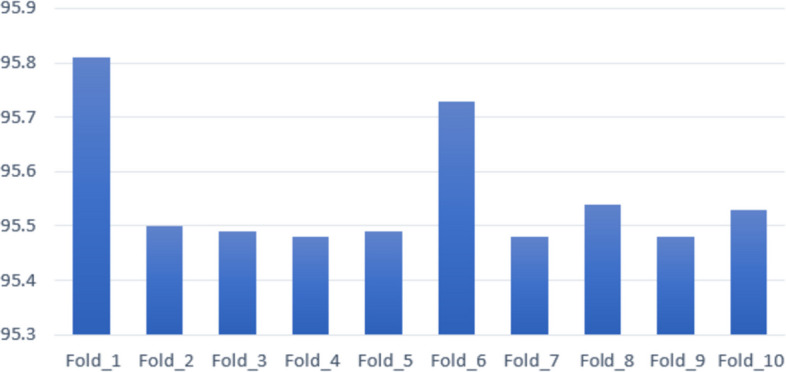
K-fold cross validation

Cross-validation is a technique used to assess a model’s capacity to accurately predict results for new, unobserved data [40]. This approach provides a more dependable estimate of the model’s performance on novel data and mitigates the risk of overfitting. The experimental results of tenfold cross-validation are shown in Fig. 15.

The model is evaluated utilizing 10 K-fold cross-validation with different *K* values ranging from 1 to 10. The *x*-axis of Fig. 15 represents the number of iterations of the cross-validation process. The *y*-axis represents the accuracy. It is found that across all folds and iterations, and the OCCT model yields over 95% test accuracy which further validates the performance consistency of the model.

### DCGAN’s Performance Comparison with Prior Studies

The performance of GAN for synthetic data generation is compared with prior studies in this section. To evaluate the quality of the generated images, we calculated the peak signal-to-noise ratio (PSNR) and structural similarity index (SSIM) for both the diabetic macular edema (DME) and Drusen classes. A total of 100 images were randomly selected for each class, comprising 50 real images and 50 synthetic images. The results, presented in Table 11, show the PSNR and SSIM values for each class, along with the mean values across both classes.

**Table 11:** the PSNR and SSIM for 100 images of DME and Drusen classes

Class	SSIM	PSNR (dB)
DME	0.7679	25.38
Drusen	0.7915	25.93
Mean	0.7794	25.66

The PSNR and SSIM values presented in Table 11 indicate the quality of the GAN-generated images compared to real images. The PSNR values are 25.38 dB for DME and 25.93 dB for Drusen, suggesting that the synthetic images closely resemble the real images in terms of signal quality. The SSIM values are 0.7679 for DME and 0.7915 for Drusen, with both values above 0.7, indicating a high level of structural similarity between the synthetic and real images. The performance of our DCGAN model is benchmarked against existing GAN models, as shown in Table 12.

**Table 12:** Comparative analysis of DCGAN’s performance with prior studies

Authors	SSIM	PSNR (dB)
Wijanto et al. [41]	0.54	25.3
Xie et al. [42]	0.95	28.1
Kande et al. [43]	0.83	28.25
Chen et al. [44]	0.88	25.45
Qiu et al. [45]	0.71	26.4
Gour et al. [46]	0.68	27.55
Our	0.7794	25.66

In the comparative analysis, though values for SSIM (0.7794) and PSNR (25.66 dB) achieved by our DCGAN model are not superior to all other studies, they are still within a range that may considered acceptable for generating high-quality synthetic images. For instance, our model outperforms the GAN models in Wijanto et al. [41] study, where both PSNR of 25.3 dB and an SSIM of 0.54 scores are lower than our DCGAN and in Chen et al. [44] study, which reported a PSNR of 25.45 dB. This demonstrates that the DCGAN model generates images of higher quality in terms of PSNR while maintaining competitive structural similarity. Moreover, our results are close to scores achieved by Qiu et al. and Kande et al. Qiu et al. [45] attained a PSNR of 26.4 dB and an SSIM of 0.71 (SSIM lower than DCGAN), whereas Xie et al. [42], Kande et al. [43], and Gour et al. [46] reported a PSNR of 28.1 dB, 28.25 dB, and 27.55 dB and an SSIM of 0.95, 0.83, and 0.68 (SSIM lower than DCGAN), respectively. Although our PSNR and SSIM values are slightly lower in some cases, they remain within a comparable range, indicating our DCGAN model’s performance is close to these strong benchmarks. It is important to note that performance metrics across different studies may vary due to differences in datasets and class distributions used. Despite these variations, the threshold values of PSNR and SSIM range mostly above 25 dB and 0.68. The values achieved by our DCGAN model fall within the benchmark thresholds, and by maintaining good PSNR and SSIM values, the DCGAN model underscores its effectiveness in producing realistic and structurally coherent images, thereby supporting robust proposed model’s training, and reducing class imbalance in the dataset.

### Proposed Model’s Performance Comparison with Prior Studies

In this section, a comparative analysis with existing studies is carried out to demonstrate the effectiveness of this study. Table 13 displays the name of authors, publication year, dataset, classes, proposed method, and its findings.

**Table 13:** Comparative analysis of proposed model’s performance with prior studies

Authors	Year	Dataset	Classes	Number of data	Method	Result (Accuracy)
Elkholy et al. [47]	2024	OCT dataset [48]	Normal, CNV, DME, Drusen	35,468	Proposed an optimized CNN model for classification	97%
Dai et al. [49]	2024	OCT dataset [50]	Normal, CNV, DME, Drusen	108,312	Utilized the ResNet50, DenseNet121, and InceptionV3 models, pre-trained on RadImageNet, enhanced with a sample replication technique	Approximately 95% for all models
Dutta et al. [51]	2023	OCT dataset [50]	Normal, CNV, DME, Drusen	109,309	Proposed a Conv-ViT model	94.46%
Huang et al. [52]	2023	OCT dataset [50]	Normal, CNV, DME, Drusen	108,31	Proposed GABNet for classification	96.5%
Paluru et al. [53]	2023	University of California San Diego (UCSD) dataset	AMD and Normal	109,300	Proposed a self-distillation framework with different baseline models including ResNet18, MobileNetV2, and ShuffleNetV2	F1 score 95% for ResNet18
Baharlouei et al. [54]	2023	1. OCTID 2. Duke 3. Heidelberg 4. TOPCON	1. Normal, CSR, MH, AMD, DR 2. Normal, DME, AMD 3. Normal, DME, AMD 4. Normal, DME	1. 572 2. 3231 5. 4254 4. 57,171	Proposed a CNN with wavelet scattering transform (WST) for classification	1. 82.5% 2. 96.6% 3. 97.1% 4. 94.4%
Özdaş et al. [55]	2023	1. Dataset 1 [56] 2. Dataset 2 [57]	1. Normal, CNV, DME, Drusen 2. Normal, DME, AMD	1. 14,568 2. 3231	Combined feature extraction methods and used the firefly algorithm with machine learning classifiers for classification	1. 95.7% 2. 95.4%
OCCT (proposed)		OCT dataset [14]	Normal, CNV, DME, Drusen	130,649 (augmented)	Proposed an effective augmentation and classification approach	97.09%

The proposed classification method offers substantial improvements in retinal disease diagnosis compared to previous studies, which reported accuracies ranging from 82 to 97%. While most of these studies typically used the same number of classes as ours, they often failed to adequately address class imbalance which affect adversely in model performance. Furthermore, there was a general lack of emphasis on optimizing models for reduced complexity. Our innovative approach not only addresses these issues but also surpasses other studies without relying heavily on a balanced dataset.

### Limitations

The research describes a novel deep learning strategy for categorizing retinal diseases, with an emphasis on optical coherence tomography (OCT) images. While the findings are promising, several limitations should be considered. The quality and diversity of the dataset used for training and the impact of data augmentation techniques, such as generative adversarial networks (GANs), on model performance should be thoroughly evaluated. The major evaluation parameter in the paper is accuracy, which may not adequately represent the clinical utility of the model. Therefore, sensitivity, specificity, and AUC-ROC assessments are required. The adaptability to new retinal conditions, as well as the requirement for longitudinal investigations, should be recognized. Finally, the study should compare its clinical value and utility in practice not only with other transformer-based models but also with existing diagnostic approaches.

## Conclusion

Artificial intelligence (AI) plays a significant role in disease diagnosis by enabling the automatic analysis of medical images and large-scale patient data. Such capability allows for the identification of intricate patterns and relationships of medical data that may not be immediately apparent to human experts. This research presents the OCCT, a deep learning model based on a transformer learning model CCT for retinal disease classification. The OCT dataset of retina is enhanced with data augmentation employing the DCGAN for reducing the class imbalance issue. The enhanced dataset is further process through several pre-processing techniques for better visualization and boost the model training. The CCT model is optimized through an ablation study creating an optimized model named OCCT and trained with the DCGAN generated enhanced dataset. The proposed OCCT model has demonstrated superior performance compared to vision transformer and Swin transformer models. The OCCT model is computationally efficient, with fewer parameters than ViT and Swin Transformer models, making it suitable for resource-constrained environments. It also outperforms eight state-of-the-art transfer learning models in terms of both accuracy and computational efficiency. *K*-fold cross-validation further validates the model’s reliability. The model maintains high accuracy even with reduced training images and shows no class bias, underscoring its robustness. In conclusion, the proposed methodology of this study offers an efficient solution for retinal disease classification with imbalanced dataset, highlighting the potential of AI in medical diagnostics and its applicability in real-world healthcare scenarios.

## Data Availability

The dataset is available and it is free at Mendeley. 10.17632/rscbjbr9sj.3
